# Digger: directed annotation of immunoglobulin and T cell receptor V, D, and J gene sequences and assemblies

**DOI:** 10.1093/bioinformatics/btae144

**Published:** 2024-03-13

**Authors:** William D Lees, Swati Saha, Gur Yaari, Corey T Watson

**Affiliations:** Bioengineering Program, Faculty of Engineering, Bar-Ilan University, Ramat Gan, 5290002, Israel; Department of Biochemistry and Molecular Genetics, School of Medicine, University of Louisville, Louisville, Kentucky 40292, United States; Bioengineering Program, Faculty of Engineering, Bar-Ilan University, Ramat Gan, 5290002, Israel; Department of Biochemistry and Molecular Genetics, School of Medicine, University of Louisville, Louisville, Kentucky 40292, United States

## Abstract

**Summary:**

Knowledge of immunoglobulin and T cell receptor encoding genes is derived from high-quality genomic sequencing. High-throughput sequencing is delivering large volumes of data, and precise, high-throughput approaches to annotation are needed. Digger is an automated tool that identifies coding and regulatory regions of these genes, with results comparable to those obtained by current expert curational methods.

**Availability and implementation:**

Digger is published under open source license at https://github.com/williamdlees/Digger and is available as a Python package and a Docker container.

## 1 Introduction 

**Figure 1.**
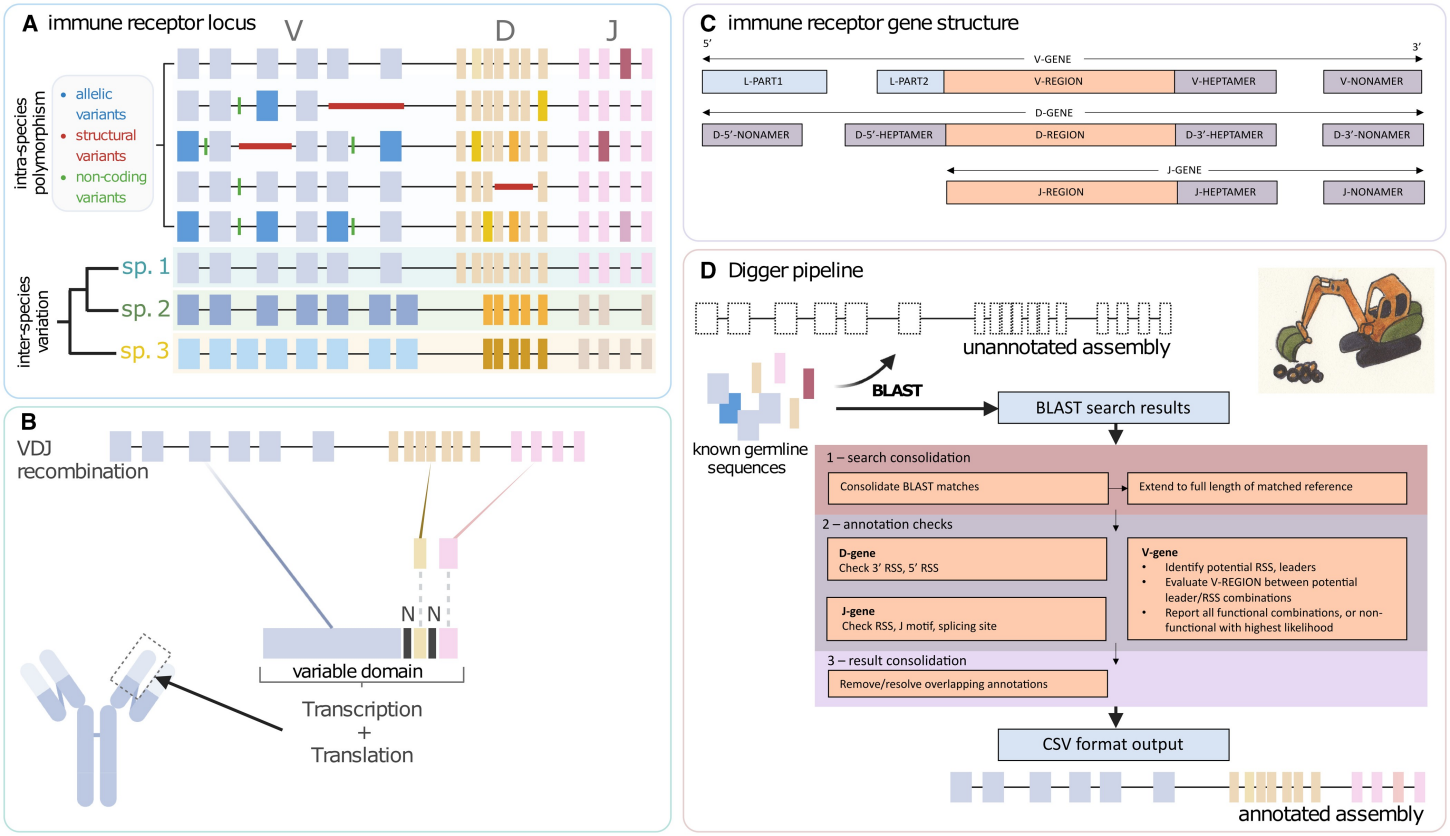
(A) A conceptual IGH locus, with receptor genes depicted as rectangles. Intra-species polymorphism can include allelic variation (shown by shading), structural variation (thicker central line), and non-coding variants (small bars). Between species, there is a wide variation in the number of functional genes and their relative location, mandating species-specific gene identification and labeling. (B) The complete gene encoding the variable domain of an IG/TR receptor is brought together from V, (D), and J segments through somatic recombination. (C) Alongside the core coding region (V-REGION, D-REGION, J-REGION), the genes contain regulatory regions associated with recombination (HEPTAMER, NONAMER) and cellular secretion (L-PART1, L-PART2). Those shown as annotated by Digger. (D) BLAST is used to identify gene locations, using a reference set of known germline sequences. After consolidation of results, locations are checked for known indicators of functionality. Sequences are further consolidated to remove duplicates. Digger graphic by Susan Lees, with permission.

Immunoglobulin receptor (IG) and T cell receptor (TR) germline variable (V), diversity (D), and joining (J) genes undergo a process of somatic recombination to form receptor encoding DNA sequences (Early *et al.* 1980, Fig. 1A). Previously undiscovered germline alleles are frequently reported even in humans, and the genes and their alleles in many other species are only partially catalogued (Vázquez Bernat *et al.* 2021, Rodriguez *et al.* 2022). More complete knowledge is of utmost importance as there is growing evidence that the germline genotype has an important bearing on the nature and outcome of immune responses (Stephen *et al.* 2023, Yuan *et al.* 2023). The full gene structure can only be determined by sequencing genes from germline genomic material. The “flanking regions” surrounding the core coding regions are essential to the formation of templates for transcription and translation of viable receptors (Fig. 1B). Based on sequence characteristics of these elements, IG/TR genes and alleles are grouped into “Functional”, “ORF”, and “Pseudogenes” using a classification and ontology codified by the International IMmunoGeneTics Information System (IMGT) (Giudicelli *et al.* 2006).

Source sequences for germline receptors can be found in GenBank (Benson *et al.* 2008), ranging from sequences of single genes to sequences of entire IG and TR loci. Many have been catalogued and annotated by IMGT. The loci contain highly similar genes in close proximity, with extensive inter- and intra-species variation (Fig. 1C). Annotation by domain experts is time-consuming. There is a pressing need for automated tools that can free experts to focus on challenging tasks. Unlike tools previously described (Lin *et al.* 2022, Sirupurapu *et al.* 2022), Digger reports flanking regions as well as coding regions, and describes features and functionality using IMGT ontology.

## 2 Materials and methods

### 2.1 Configuration

Digger requires a set of known core coding region allele sequences, commonly available in receptor germline reference sets (Collins *et al.* 2024). Position-weighted matrices (PWMs) are required for recombination signal sequence (RSS)nonamers and heptamers, and for V gene leader part 1 and part 2. PWMs for the seven human loci and for rhesus macaque IG loci are provided in the package, and the documentation describes how these can be constructed for other loci. Optionally, consensus motifs can be defined for RSS motifs.) 

### 2.2 Identification of candidate sequences

The known allele sequences are compiled into BLAST databases and blastn is run to identify matches. The search for V-sequences uses the parameters gapopen 5, gapextend 5, penalty -1, word_size 11. For D and J searches, word_size is reduced to 7 to reflect the shorter sequences, and for D sequences the evalue is set to 100 rather than the default of 10 to widen the search. These parameters were found optimal in testing but may be varied through the use of individual underlying tools if required. BLAST results at each location in the assembly are consolidated into a summary table. The best match at each position in the assembly is determined, taking account of the evalue and the proportion of the allele sequence that was matched, preferring longer matches. These best matches are extended to cover the full length of the matching sequence, and taken forward as candidates to annotate, using steps dependent up on the gene type of the matched sequence.

### 2.3 Processing and annotation of candidate sequences

*V genes*: a window at the 3′ end of the match is searched to determine the most likely location of the RSS, considering the joint likelihood of PWMs for nonamer and heptamer. The 3′ end of the V-REGION is adjusted to meet this location if necessary. This approach is then repeated at the 5′ end, considering the joint likelihood of PWMs for L-PART1 and 2. Combinations of L-PART1 and 2 that splice to form an in-frame sequence with no STOP-CODONs are preferred, as are those that match the start of the matched V-REGION. Functionality is determined as detailed in Table 1. Functional alleles are taken forward for annotation: if none, the non-functional sequence with highest leader and RSS joint likelihood is taken forward.

**Table 1.** Functionality criteria.

Type	Functionality criteria
V	RSS nonamer and heptamer pass PWM thresholds, and match canonical consensus, if defined. L-PART1 and L-PART2 pass PWM thresholds and splice to form a coding sequence with no STOP-CODONs that is in-frame with the V sequence. V-REGION is in-frame and has first and second cysteines at the correct positions in the IMGT alignment. No STOP-CODONs are present in the V-REGION before the second cysteine.
D	RSS nonamers and heptamers match canonical consensus, if defined.
J	RSS nonamer and heptamer pass PWM thresholds, and match canonical consensus, if defined. The J-motif is found at the expected position relative to the end of the J sequence. The donor splice is found at the expected position, given the length of the matched sequence.

*J genes*: RSS is determined as above. Provided that at least the nonamer or heptamer passes the PWM threshold, the sequence is taken forward.

*D genes*: 3′ and 5′ RSS are determined as above. Sequences are taken forward if both nonamers and heptamers are below the PWM threshold (the RSS criterion is more rigorous because no other features can be checked).

### 2.4 Annotation

V and J sequences that do not meet all the criteria are annotated as open reading frame (ORF) provided that they do not contain STOP-CODONs, otherwise as pseudogenes. D sequences are annotated as functional if all RSS nonamers and heptamers match the optionally defined canonical consensus, if present, otherwise as ORF. Annotations are reported in CSV format. The report includes sequences and coordinates of all discovered elements (positions in the assembly being annotated), allele functionality, the closest sequence match in any provided reference set, and annotation notes. A full description of fields is given in the documentation.

### 2.5 Consolidation

Overlapping sequences may be annotated. All sequences annotated as functional are retained, and a note is added if they overlap (this is seldom seen in practice). Any sequence annotated as non-functional that overlap with a functional sequence is discarded. If multiple non-functional sequences overlap, the sequence with highest joint likelihood of included motifs is retained and the others discarded.

## 3 Results

We compared Digger’s annotations to five IMGT-curated loci from the human reference assembly GRCh38.p12 (Guo *et al.* 2017) and the three IG loci from the rhesus macaque reference assembly rheMac10 (Warren *et al.* 2020, Nguefack Ngoune *et al.* 2022). The human light chain loci of GRCh38.p12 could not be included in the comparison as IMGT have not published locus-wide annotations. Scripts to reproduce the comparison are provided. Annotation of the human IGH locus (the most complex) completed on a 2 GHz workstation in 6 min. Across the five loci, a total of 511 alleles were classified as functional in at least one annotation (Digger or IMGT). Of these, 472 were classified as functional in both annotations, while 36 were classified as functional in one annotation and ORF in the other. Three D-alleles were identified by IMGT but not by Digger (one human T cell receptor D chain allele and two macaque Immunoglobulin heavy chain (IGH) alleles): these were the only functional alleles identified in one annotation which were not identified in the other as either functional or ORF. We address this disagreement in Section 4. The two approaches are in good agreement overall in the identification of functional alleles, with the large majority of disagreements arising from different definitions of functionality.

IMGT sometimes uses expression and other data to inform annotation: for example, some sequences were characterized as ORF by IMGT rather than functional because they had not been observed in repertoires. These classifications are considered “non-annotational” as they use information external to the sequence. IMGT annotations also contained two genes that were in reverse sense compared to other genes in the locus: these are also considered “non-annotational”, in that Digger only annotates genes that are found in a single sense (where desired it can be run twice so that genes in both senses are captured, but in this comparison that was only conducted for IGK loci). Of the 36 cases where one annotation identified an allele as functional and the other identified it as ORF, 9 were the result of non-annotational differences. Of the remaining 27, 13 arose from different characterization of the leader, 9 from different characterization of the RSS, and 5 from variation in the treatment of STOP-CODONs identified after position 104 in the V-REGION, or before the J-MOTIF in the J-REGION. In these cases, a productive rearrangement may be found if one or more of the nucleotides of the STOP-CODON are excised during junction formation. Digger allows functional alleles to contain such STOP-CODONs. IMGT excludes some examples (e.g. human TRAJ55*01, macaque IGKV2-103*01), and may be relying on expression information to guide classification.

To test the limits of annotation, we annotated the human IGH locus using rhesus macaque and mouse germline sets from IMGT as reference. Digger identified the same set of F and ORF genes with the rhesus set as with the human set. With the mouse set, V and J gene F and ORF results were identical, but 11/35 IGH D genes were not identified.

## 4 Discussion and conclusions

We have demonstrated that Digger produces annotations of gene features in functional alleles in close agreement to those produced by expert human annotators. In the ∼5% of cases where the two approaches provided a different classification of the allele on the basis of its annotation, the difference was relatively minor. There is not, at present, a clear set of universally accepted criteria for categorization of functionality or a systematic understanding of the impact of leader and RSS variation on expression and receptor functionality. With increasing volume of sequencing, greater insights can be expected. Automated tools such as Digger can enforce standardized annotation criteria.

Three D genes were classified as functional by one approach but not identified by the other. Identification of D genes through directed approaches is challenging, given that a D-REGION may be as short as 8 nt. The largest area of disagreement between the approaches was analysis of the leader. As leaders can be found in 5′ RACE repertoires (Mikocziova *et al.* 2020, Huang *et al.* 2021), it is anticipated that further study can provide insights into the impact of, for example, variation in the length of the V-SPACER, a non-canonical splicing sequence, and a non-canonical L-PART2 length.

Digger was used to annotate 962 genomic sequences supporting Adaptive Immune Receptor Repertoire Community (AIRR) human IG germline sets (Collins *et al.* 2024). This resolved a number of sequences previously reported with truncated 3′ or 5′ ends. For example, IGLV2-8*03 was reported in Berek *et al.* (1997) as a 274 nt V-sequence, currently represented in the IMGT system with a 5′ 23 nt truncation. BLAST search identified an assembly (GenBank accession CP068256) which was annotated to provide the full-length sequence. Digger can support the challenge posed by the ever-increasing volume of genomic sequencing. It facilitates revision of annotations as the classification system is refined. We have demonstrated its use with reference sequences from another species, where good results can be obtained with even a distant species, although some manual curation may be expected for complete coverage.

## Data Availability

Source code, documentation, and annotations of loci described in this paper are at https://github.com/williamdlees/Digger. Annotations of the human light chain loci from GRCh38.p12 are also provided. Digger requires Python (v3.9+) and a locally-installed copy of BLAST (v2.14+) (Altschul *et al.* 1990).
